# Pathological correlates of impaired self-awareness of memory function in Alzheimer’s disease

**DOI:** 10.1186/s13195-021-00856-x

**Published:** 2021-06-25

**Authors:** Geoffroy Gagliardi, Madeline Kuppe, Cristina Lois, Bernard Hanseeuw, Patrizia Vannini, for the Alzheimer’s Disease Neuroimaging Initiative

**Affiliations:** 1 Brigham and Women’s Hospital, Boston, MA USA; 2 Massachusetts General Hospital, Boston, MA USA; 3 Harvard Medical School, Boston, MA USA

**Keywords:** Alzheimer’s disease, Awareness, Cognition, Tau, Amyloid

## Abstract

**Introduction:**

Impaired self-awareness of memory function, a.k.a. anosognosia, is a common symptom in Alzheimer’s disease (AD); however, its pathological correlates remain unclear. Here, we investigated the impact of amyloid and tau on memory self-awareness.

**Methods:**

Two hundred thirty-six clinically normal (N) and 102 impaired (I) participants from the ADNI cohort were included. Amyloid (global) and tau burden (in entorhinal and inferior temporal cortices) were assessed using positron emission tomography (PET). Self-awareness of memory was assessed using discrepancy indexes of subjective participant-informant ratings, as well as participant-objective scores of memory performance. Subjective and objective values were derived from the Everyday Cognition memory questionnaire and Logical Memory (delayed recall).

**Results:**

Lower awareness (both methods) of memory function was associated with higher levels of pathology in the I group as compared to N. There was a significant effect of tauopathy, but not amyloidosis, on individual complaint, such that higher levels of tau associated with lower awareness.

**Discussion:**

Impaired self-awareness appears progressively in the evolution of the disease related to AD biomarkers. Discordant subjective and objective measures may be important for clinical consideration.

**Supplementary Information:**

The online version contains supplementary material available at 10.1186/s13195-021-00856-x.

## Introduction

Coined by Joseph Babinsky at the beginning of the twentieth century, the term anosognosia originally described unawareness of hemiplegia after a stroke [1]. Since then, this concept has expanded to include lack of awareness for a cognitive, behavioral, or functional impairment [2, 3]. Anosognosia is a common symptom in the dementia stage of Alzheimer’s disease (AD), and it has been estimated that between 20 and 80% of patients suffer from some form of unawareness, with a progressive increase in this prevalence with time [4]. AD is a neurodegenerative disease in which behavioral and cognitive changes occur over decades. Previous research has suggested that, starting at the preclinical stage, an individual may experience heightened awareness of changes in his/her cognition that cannot be measured with clinical tests and/or detected by people around them [5, 6]. Also known as subjective cognitive decline (SCD) [7], this phenomenon has been widely studied and relates to an increased risk of future cognitive decline [8]. For instance, cognitively normal participants with SCD have been shown to have a higher risk of conversion to mild cognitive impairment (MCI) as well as AD dementia [9–13]. In contrast, recent studies have found that lack of awareness of cognitive changes, rather than SCD, predicts progression from the asymptomatic stage to MCI [5]. Here, lack of awareness was defined by an informant complaining significantly more than the participant. Although existing literature describes three primary methods of measuring awareness in AD patients, all of them consist of a comparison between a control measure and the subject’s perception of his/her own cognitive functioning [4, 14, 15]. While the use of the participant’s subjective self-rating is constant across methods, the control measure can be the participant’s objective cognitive performance (participant-objective discrepancy), an informant’s perception (participant-informant discrepancy), or a clinician’s assessment. While currently there is no consensus on how to optimally assess self-awareness of memory, it is important to recognize that while these methods use slightly different approaches, an overlap has been observed when comparing results across studies [15]. All in all, this has led to the idea that unawareness may start to evolve earlier than previously thought, i.e., during the prodromal [16–20] and even the preclinical stages of AD [21–23]. In fact, to date, three longitudinal studies have demonstrated that lack of awareness of memory impairment appears approximately 2 to 3 years before the clinical progression to AD diagnosis [24]. Of these studies, Wilson and colleagues [24] used the participant-objective discrepancy method, while Hanseeuw et al. [5] and Vannini et al. [25] used the participant-informant discrepancy. Despite these consistent findings, what is less clear is the mechanism underlying altered self-awareness across the AD spectrum (i.e., from the preclinical to the dementia stage), and especially any relationship between self-awareness and AD pathology.

AD is characterized by a specific pattern of lesion accumulation over time, the two pathological hallmarks being amyloidosis and tauopathy [26]. With the development of positron emission tomography (PET), both of these biomarkers can now be measured in vivo and have been shown to accumulate decades before a clinical diagnosis of AD dementia can be made [27, 28]. This has led to the definition of AD as a continuum, and the proposal of the prodromal and preclinical stages in which the clinical symptoms are not yet sufficient to have a functional impact on cognitive tests and activities of daily living [26, 29, 30]. Previous studies have also demonstrated a relationship between biomarker accumulation and cognitive decline, and this relationship has been found to exist years before a clinical diagnosis of AD dementia [31–33]. Similarly, previous studies investigating amyloidosis in particular have found that participants with altered awareness—either heightened [34–36] or reduced [22, 23]—harbor higher levels of amyloid burden in their brain. Loss of awareness of the cognitive decline in AD has also been associated with hypometabolism (as assessed using FDG-PET) [4, 23], and network dysfunction (as assessed using resting state functional magnetic resonance imaging), in a set of brain regions commonly referred to as the Default Mode Network (DMN). Thought to be involved in self-referential processing [37], these brain regions have also been shown to be affected early both by amyloid and tau in AD [38], leading to the idea that loss of awareness might be the result of increased AD pathology. Although this seems to be the case with regard to amyloid, the relationship between tau and awareness across the AD continuum is not known.

Thus, in this study, we aimed to investigate the impact of amyloid and tau accumulation on memory self-awareness across the AD spectrum. Specifically, we used a cohort of both clinically normal and impaired participants, all of whom had in vivo PET imaging that assessed amyloid and tau, as well as a validated participant-informant questionnaire and objective memory performance to measure awareness of cognition using two approaches. Consequently, two awareness measures were derived via a discrepancy score between the participant and informant complaints, as well as a discrepancy score between the participant’s complaints and an objective memory test, allowing examination of the effect of biomarkers on awareness. We hypothesized that altered awareness would be related to AD pathology, such that increased pathology would be associated with heightened awareness in cognitively normal (N) participants, while the same would be associated with decreased awareness in clinically impaired (I) participants.

## Methods and materials

### Population

Data used in the preparation of this article were obtained from the Alzheimer’s Disease Neuroimaging Initiative (ADNI) database (adni.loni.usc.edu). The ADNI is an ongoing, longitudinal, multicenter study conducted at 59 sites across North America, enrolling CN, amnestic MCI, and AD participants aged 55 to 94 years. The ADNI was launched in 2003 as a public-private partnership, led by Principal Investigator Michael W. Weiner, MD. The primary goal of ADNI has been to test whether serial magnetic resonance imaging (MRI), PET, other biological markers, and clinical and neuropsychological assessment can be combined to measure the progression of MCI and early AD. For up-to-date information, see www.adni-info.org.

Each participant in the study (*n* = 338) had PET scan data using ^18^*F* − *AV*45 (amyloid tracer) and AV1451 (tau tracer), Everyday Cognition (ECog) scores for both participant and informant, and a Clinical Dementia Rating (CDR) score [39]. All participants underwent comprehensive neuropsychological assessment, including the Mini-Mental State Examination (MMSE) [40], Rey Auditory Verbal Learning Test (RAVLT) [41], and Logical Memory [42], Categorical Fluency [43], Trail Making Test [44], and the Geriatric Depression Scale (GDS) [45]. Using global CDR as a criterion, we identified 236 participants clinically normal (N, CDR = 0 and MMSE > 24) and 102 as clinically impaired (I; CDR ≥0.5).

Demographic characteristics are summarized in Table 1.

**Table 1:** Demographics characteristics and group comparisons

	All	Normal		Impaired		Clinical groups	
		A−	A+	A−	A+	Normal	Impaired
N	338	151	85	50	52	236	102
Age	71.42 (6.53)	69.94 (5.64)	73.11 (6.59) a**	71.53 (7.78)	72.88 (6.69) a*	71.08 (6.18)	72.21 (7.25)
Gender	174F (51.48%)	88F (58.28%)	47F (55.29%)	17F (34%) a**	22F (42.31%)	135F (57.2%)	39F (38.24%) c**
Education (years of study)	16.69 (2.61)	16.97 (2.48)	16.79 (2.39)	15.98 (3.09)	16.4 (2.72)	16.91 (2.44)	16.2 (2.9)
APOE4 status (E4)	66 (19.53%)	18 (11.92%)	25 (29.41%) a**	8 (16%)	15 (28.85%) a**, b*	43 (18.22%)	23 (22.55%) c*
MMSE	28.81 (1.41)	29.25 (1.08)	28.68 (1.44) a**	28.48 (1.42) a**	28.06 (1.76) a**	29.04 (1.25)	28.26 (1.61) c**
GDS	1.4 (1.85)	1.14 (1.74)	1.04 (1.19)	1.74 (2.19)	2.14 (2.27)	1.1 (1.54)	1.93 (2.22) c*
Logical Memory (delayed)	12.51 (4.8)	13.85 (3.88)	14.33 (3.7)	11.14 (4.38) a**	6.98 (4.77) a**, b**	14.02 (3.82)	9.02 (5.01) c**
Categorical fluency	20.43 (5.63)	21.86 (5.27)	21.19 (5.57)	18.98 (4.93) a**	16.43 (5.26) a**	21.62 (5.38)	17.69 (5.23) c**
TMT B-A time	86.69 (51.35)	68.66 (25.09)	82.06 (39.72) a*	105.58 (64.47) a**	128.12 (76.56) a**	73.51 (31.77)	116.96 (71.38) c**
SUVr tau (flortaucipir, AV1451)—entorhinal cortex	2.08 (0.65)	1.85 (0.37)	2.06 (0.5) a**	2.02 (0.64)	2.84 (0.93) a**, b**	1.93 (0.43)	2.44 (0.89) c**
SUVr tau (flortaucipir, AV1451)—inferior temporal cortex	2.06 (0.52)	1.9 (0.19)	2.05 (0.33) a**	2.01 (0.22) a*	2.63 (1.03) a**, b**	1.95 (0.26)	2.32 (0.81) c**
Amyloid (florbetapir-AV45)	1.15 (0.22)	1.01 (0.05)	1.31 (0.17) a**	0.99 (0.07) a*	1.43 (0.21) a**, b**	1.12 (0.18)	1.21 (0.27) c**
Informant complain (ECog memory)	1.61 (0.67)	1.34 (0.42)	1.39 (0.46)	2.07 (0.65) a**	2.34 (0.81) a**	1.36 (0.43)	2.21 (0.75) c**
Participant complain (ECog memory)	1.87 (0.67)	1.6 (0.49)	1.82 (0.61) a*	2.25 (0.74) a**	2.38 (0.71) a**	1.68 (0.54)	2.32 (0.72) c**
Awareness (participant-informant)	0.26 (0.69)	0.26 (0.51)	0.43 (0.62)	0.19 (0.77)	0.04 (1.05)	0.32 (0.56)	0.11 (0.92)
Awareness (objective-subjective)	−0.04 (1.44)	−0.19 (1.27)	0.34 (1.39) a*	0.3 (1.47)	−0.56 (1.74) b*	0 (1.34)	−0.14 (1.66)

*Note*: *A+/−* amyloid status (positive/negative, threshold = 1.11), *APOE* apolipoprotein, *MMSE* Mini-Mental State Examination, *GDS* Geriatic Depression Scale, *RAVLT* Rey Auditory Verbal Learning Test, *ECog* Everyday Cognition; a, vs normal A−; b, vs impaired A−; c, vs normal. *< 0.05, **< 0.001

### Awareness of memory

Two types of awareness indexes were computed. First, awareness of memory function was assessed using the ECog scale [46], which is a 39-item questionnaire collecting subjective ratings from both participants and informants, in which questions are framed to compare the participant’s current cognitive functioning to an estimation of their performance 10 years ago. The responses are measured on a Likert scale from 1 (Better or no change) to 4 (Consistently much Worse), with a higher score indicating a perceived decline in cognition.

An awareness index was obtained by calculating a discrepancy score between the participant and the informant report on the 8-item memory subscale of the ECog scale [5]. Subsequently, a negative awareness index would suggest that the participant is over-estimating his/her memory as compared to the informant, and a positive awareness index would suggest that the participant is under-estimating his/her memory as compared to the informant. An awareness index score close to 0 would indicate that the participant and informant are in accord.

An additional awareness index was computed by comparing the participant’s objective memory performance with his/her subjective self-rating. As above, ECog memory was used for the subjective aspect, while objective memory was assessed with the Logical Memory delayed recall score. Following previous methods [6], both scores were z-transformed using the mean and standard deviation of participants in the cognitively normal group. ECog z-scores were inverted in order to match the memory scores (i.e., with higher scores indicating better performance). Finally, a discrepancy score was then calculated between objective and subjective performances. Same as above, an awareness index score close to 0 would indicate a good match between the measures, while positive and negative scores would suggest over- and under-estimation, respectively.

### Imaging

Amyloidosis was measured with PET and florbetapir (^18^F-AV45) as a tracer. Standard uptake value ratio (SUVr) was computed using the whole cerebellum as a reference to estimate “global” amyloid burden. Participants were also classified as amyloid positive (A+) or negative (A−) using a 1.11 threshold [38].

Tauopathy was measured with PET and flortaucipir (^18^F-AV1451) as a tracer. SUVr were computed from two regions of interest (ROI), the entorhinal (EC) and the inferior temporal (IT) cortices, using the inferior cerebellar cortex as a reference, and partial-volume correction (PVC, using a geometric transfer matrix approach). These regions were selected as they are affected early in the course of AD (for EC) [39, 40] and thus are widely considered characteristic of AD pathology rather than normal aging (IT) [41].

### Statistical analyses

Four subgroups were created based on clinical status (i.e., normal [N] vs impaired [I]) and amyloidosis (i.e., A+ vs A−). We defined NA− participants as a “reference” group. Two-sample t-tests and chi-squared tests were performed to examine the differences in demographic, behavioral (both objective and subjective), and biologic (amyloid and tau) measures. Three sets of comparisons were made (i) comparing each group versus the reference group (i.e., NA−), (ii) comparing A+ vs A− groups across the clinical categories (i.e., I and N), and (iii) comparing clinically normal and impaired participants (irrespective of amyloid status).

To assess the influence of biomarkers, and in particular tau, on awareness level, we performed a linear regression model with the awareness index as the dependent variable. The demographics (i.e., age, gender, and number of years of education) were added as covariates, while clinical status (i.e., N vs I), amyloid (^18^F-AV45 PET SUVr), and tau burden (^18^F-AV1451 PET SUVr) were included as independent variables. The model also assessed for interactions (two- and three-way) between these independent variables. The final model was as follows: *Awareness* ∼ *Covariates* + *Amyloid* × *Clinical Status* × *Tau*. We performed separate models for the EC and IT tau SUVr values, using our two different awareness indexes (i.e., participant vs informant and *participant vs objective*). Two models were performed to assess the EC and IT tau SUVr values separately. We computed the type II likelihood ratio to test the main and interaction effects and Cohen’s f^2^ to estimate the effect size. Simple slope analyses (SSA) [47, 48] were conducted to assess the effects of both biomarkers on awareness in the two clinical (i.e., N and I) groups. For this analysis, amyloidosis was then considered at moderate, high, and low levels using the mean and the standard deviation. Interactions were investigated using the interaction R package [49]. In addition, we conducted supplementary models to investigate the influence of education on the results but adding an interaction term between biomarkers and educational level (see Supplementary Table 1).

All presented p-values were corrected for multiple comparisons using the Benjamini-Hochberg method. Graphical visualizations were performed computing estimated marginal means [50]. All statistical analyses were performed using R 3.6.3 (https://www.R-project.org/).

## Results

### Group comparisons

Of our 236 clinically normal individuals, 151 were classified as A− and 85 as A+. In the clinically impaired group, 50 were classified as A− and 52 as A+ (see Table 1). When comparing baseline information of the clinically normal and impaired participants, we observed that the clinically impaired group had significantly fewer females (*p* < 0.01), but groups did not differ in age or level of education (both *p* > 0.05). Furthermore, impaired participants demonstrated lower performance on all neuropsychological tests, as well as higher levels of depression (all *p* < 0.01). The impaired group consisted of more APOE4 carriers and had higher levels of AD biomarkers (both tau and amyloid; all *p* < 0.01). Finally, both participants and informants expressed higher levels of complaint in the impaired group (both *p* < 0.01), but no significant differences were observed regarding the awareness indexes (both *p* > 0.05).

When looking at the four different subgroups, as compared to our reference group (NA−), we observed that the IA− group included fewer females (58.28% and 34%, respectively, *p* < 0.05). No significant difference was observed between groups in the level of education or depression scores (all *p* > 0.05). Amyloid-positive participants, both normal (NA+) and impaired (IA+), were also older than the NA− group (both *p* < 0.05). There was no significant difference between the low amyloid groups (i.e., NA− vs IA−) for apolipoprotein (APOE4) carriers; however, there were significantly more APOE4 carriers in the NA+ and IA+ groups as compared to the NA− and IA− groups. The reference group (NA−) had a higher MMSE score (mean = 29.25) as compared to the other groups (all *p* < 0.001). The same pattern was observed for the Trail Making Test B-A time score, with NA− participants showing better performances than the other groups. Regarding memory (Logical Memory Delayed) and Categorical Fluency, no significant differences were observed between the clinical normal groups. However, the impaired groups were significantly less efficient than the reference group, with the IA+ participants showing lower performances than the IA−. No significant differences were observed for informant complaints between the NA− and NA+ groups as well as between IA− and IA+ groups. However, the NA+ participants complained significantly more than the NA− participants (*p* < 0.05). The clinically impaired (IA−, IA+) groups demonstrated significantly more complaints (both partner and participant) as compared to the reference group (NA− < [IA− = IA+], *p* < 0.001). No significant difference was observed between groups for the awareness indexes (all *p* > 0.05).

With regard to the biomarker measures, A+ and A− groups demonstrated, by definition, significantly different values of amyloid tracer uptake. For measures of tau tracer uptake in both NA+ and IA+ groups, significantly higher values were observed in both regions (i.e., EC and IT) compared to the reference group (*p* < 0.001), while the IA− group demonstrated this difference in the IT only (*p* < 0.05). Compared to the IA− group, the IA+ group also demonstrated significantly higher tau tracer uptake in both EC and IT regions (*p* < 0.001).

### Biomarkers’ effect on awareness

Linear regression models with the awareness index as a dependent variable, and biomarkers as predictors, were used to investigate the AD biomarkers’ effect on awareness.

First, we conducted models predicting awareness calculated as the difference between participant and informant levels of complaint (participant-informant discrepancy index). Two models were performed, using PET tau uptake from EC and IT (R2 = 0.11 and 0.12, respectively). The details of the models are presented in Table 2. Results did not demonstrate a significant effect of demographics (i.e., age, gender, and education) on the level of awareness. No significant main effect of clinical status or amyloid on the level of awareness was observed (both *p* > 0.05). Although tau burden in the EC and IT was significantly related to awareness (both *p* < 0.05), such that more tracer uptake was related to higher awareness index values overall, interpretation of these results is limited due to significant interactions.

**Table 2:** Relationship between awareness and AD biomarkers

	Participants-informants								Objective-subjective							
	SUVr tau entorhinal			SUVr tau inferior temporal					SUVr tau entorhinal			SUVr tau inferior temporal				
Vars	Estimate	SD	ESs	P	Estimate	SD	ESs	P	Estimate	SD	ESs	P	Estimate	SD	ESs	P
(Intercept)	0.38	0.47			0.52	0.47			0.71	0.97			0.75	0.99		
Age	0	0.01	0	1.000	0	0.01	0	1.000	−0.02	0.01	0.011	0.090	−0.02	0.01	0.009	0.115
Gender	−0.13	0.08	0.008	0.200	−0.12	0.08	0.007	0.245	−0.27	0.16	0.009	0.192	−0.27	0.16	0.008	0.208
Education	0	0.01	0	1.000	0	0.01	0	1.000	0.07	0.03	0.015	0.058	0.06	0.03	0.012	0.101
Clinical status			0	0.787			0.003	0.249			0.006	0.574			0.002	1.000
Clinical StatusImpaired	−0.04	0.09			−0.09	0.09			0.26	0.19			0.15	0.19		
Amyloid	0.12	0.05	0	0.927	0.11	0.05	0	0.659	0.36	0.11	0	0.583	0.37	0.12	0	0.577
Tau	−0.07	0.07	0	0.037*	−0.02	0.1	0	0.023*	−0.17	0.14	0	0.081	−0.23	0.21	0	0.243
Clinical Status:Amyloid			0.004	0.201			0.005	0.408			0.014	0.019*			0.032	0.003*
Clinical StatusImpaired:Amyloid	−0.1	0.09			−0.1	0.08			−0.4	0.18			−0.58	0.18		
Clinical Status:Tau			0	1.000			0.002	1.000			0.001	1.000			0.004	0.704
Clinical StatusImpaired:Tau	0.02	0.09			0.09	0.13			0.12	0.19			0.31	0.27		
Amyloid:Tau	0.11	0.07	0	0.086	0.11	0.09	0	0.004*	0.17	0.15	0	0.087	0.07	0.2	0	0.347
Clinical Status:Amyloid:Tau			0.023	0.013*			0.015	0.057			0.016	0.046*			0.002	0.839
Clinical StatusImpaired:Amyloid:Tau	−0.22	0.08			−0.22	0.1			−0.38	0.17			−0.17	0.21		

*Note*: *SD* standard deviation, *ESs* effect sizes (Cohen’s f2), *Amyloid* florbetapir PET SUVr, *Tau* flortaucipir PET SUVr

**Fig. 1:**
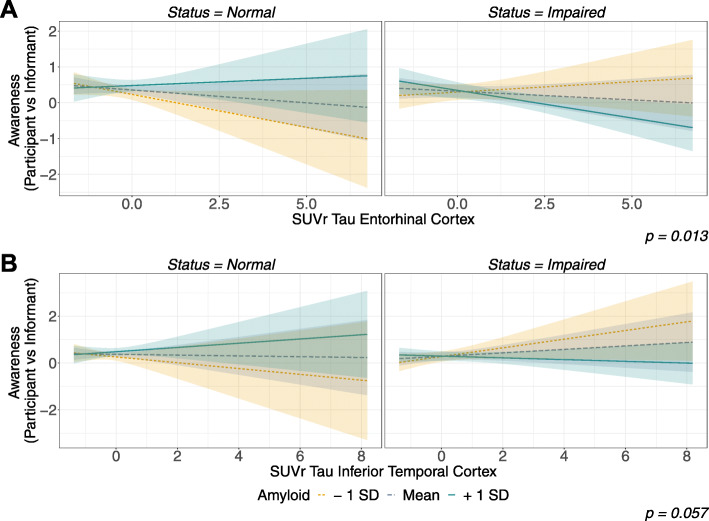
AD biomarkers’ influence on awareness (participant vs informant) among clinical stages. Notes: This figure presents the projected values of awareness within the different clinical groups (i.e., normal and impaired on the left and right panels, respectively) as predicted by both Tau SUVr (**A** entorhinal cortex, **B** inferior temporal cortex) and amyloidosis (for low, medium, and high levels of amyloid burden; represented in yellow, gray, and green, respectively). Results from the entorhinal cortex (line A) showed a significant interaction between both biomarkers (p = 0.013), with amyloid-negative clinically normal participants showing a lower awareness associated with greater tau burden (p = 0.06). In the impaired group, the opposite pattern was observed with a significant negative relationship for the participant with higher amyloid burden (p = 0.01). The model using the inferior temporal tau burden showed only a trending interaction (p = 0.057)

Specifically, the IT model demonstrated a significant interaction between AD biomarkers (i.e., amyloid and tau; *p* = 0.004), suggesting that lesser awareness may in fact be associated with greater pathology. While no significant interaction effect was found between clinical status and each AD biomarker (i.e., status × amyloid and status × tau), a significant three-way interaction was found in the EC model between AD biomarkers and clinical status (*p* = 0.013; see Fig. 1). Post hoc analyses, using SSA, revealed a negative relationship (β = −0.28, S.E. = 0.15, *p* = 0.06) between awareness and tau in the NA− group (see yellow lines in Fig. 1, left panels), such that lower scores on the awareness index were associated with higher levels of tau. In the clinically impaired (i.e., I) group, we did not observe significant slopes between awareness and tau in the EC for low and moderate levels of amyloid (Fig. 1A, right panel, yellow and blue lines); however, a significant slope for higher levels of amyloidosis was observed (Fig. 1A, right panel, green line). That is, with higher levels of tau burden, lower levels of awareness were observed (β = −0.24, S.E. = −2.73, p = 0.01). In the IT model, this three-way interaction reached trend level (*p* = 0.057). The SSA demonstrated that all slopes were non-significant from 0, but revealed a trend for the impaired group with lower amyloid burden (β = 0.35, S.E. = 1.76, *p* = 0.08), such that higher values of tau were associated with greater awareness.

**Fig. 2:**
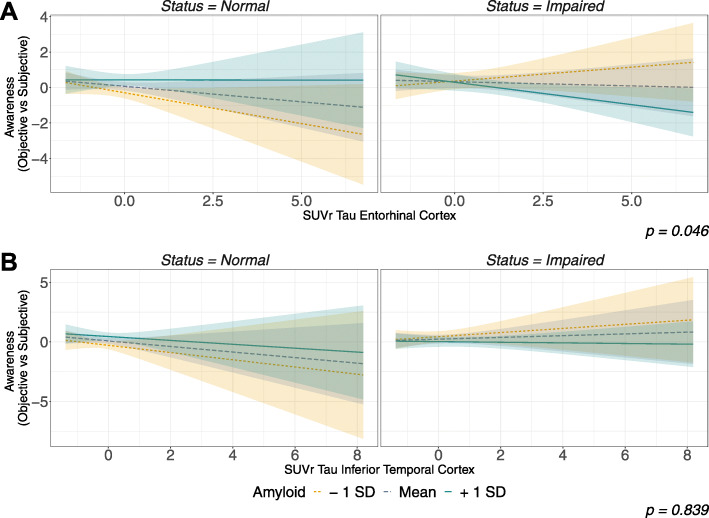
AD biomarkers’ influence on awareness (objective vs subjective) among clinical stages. Notes: Similar to Fig. 1, this figure shows projected values of awareness and its relationship to clinical status, amyloidosis, and tauopathy, using a comparison between participant’s objective (logical memory delayed recall score) and subjective (ECog memory score) performances (both being z-transformed based on normal group’s mean and standard deviation). The same codes are used for our variables of interest, i.e., normal participants being presented in the left panels while impaired can be seen on the right; entorhinal cortex tau SUVr values being displayed on the upper line, and inferior temporal cortex on the lower one; and amyloidosis being split in low, medium, and high levels of amyloid burden, represented in yellow, gray, and green, respectively. While we did not observe a significant three-way interaction for the IT model (p = 0.839), we did for the EC model (p = 0.046) in which participants from the clinically impaired group with both high amyloid and tau burden demonstrated a significantly lower awareness (β = −0.25, S.E. = 0.12, p = 0.03, upper left panel, green line) while, at lower levels of amyloid burden, we did not observe this impact. In the normal group, this interaction did not seem to be significant

Second, we conducted similar models as described above but using awareness calculated as the difference between objective and subjective ratings of memory (participant-objective discrepancy index). Similarly to the models using the participant-informant discrepancy index, we did not observe significant effects of demographics, clinical status, or amyloid burden. Furthermore, we did not observe a significant effect of tau burden (both *p* > 0.05). Finally, as described above, a similar pattern was observed with a significant three-way interaction between clinical status, amyloid, and tau burdens in the EC model (*p* = 0.046; see Figs. 1 and 2), but not in the IT model (*p* = 0.839). Post hoc analyses, using SSA, revealed a negative relationship. Specifically, in the impaired group, higher levels of both amyloidosis and tauopathy were associated with lower levels of awareness (β = −0.25, S.E. = 0.12, p = 0.03)—while low and moderate levels of amyloidosis did not demonstrate this significant relationship. In contrast, no significant relationships were observed in the normal group.

## Discussion

The current study assessed the effect of AD hallmark pathology, i.e., amyloidosis and tauopathy, on two measures of awareness of memory decline in clinically normal and impaired participants. We found a differential effect of biomarkers on awareness in the two clinical groups, such that higher levels of AD pathology were associated with lower awareness index scores (i.e., lower awareness) in the clinically impaired group as compared to that of clinically normal. Although the clinically normal group demonstrated the opposite relationship, these results were not statistically significant. Surprisingly, and in contrast to our hypothesis, we did not observe greater awareness in clinically normal participants with high levels of amyloid, although for high levels of tau (and low amyloid), a significantly lower awareness was observed.

Our sample included both clinically normal and impaired participants. In line with previous AD studies, we found that individuals with increased amyloid burden were older and included more APOE E4 carriers [27–29]. In contrast to previous studies, we did not find any differences in the level of depression (i.e., GDS score) between the groups. That is, previous studies have shown that the level of complaint can be influenced by psychiatric elements, such as anxiety or depression, and may even be the root of SCD [7]. Even though the N group was considered clinically normal based on CDR (= 0) and MMSE (≥ 24/30), we found that the NA− group had higher MMSE scores as compared to the NA+ group, suggesting that amyloid positivity in the absence of clinical symptoms may associate with poorer cognitive efficiency.

In this study, awareness was measured using a participant-informant as well as a participant-objective discrepancy score [4]. In previous studies, awareness indexes (using both approaches) have been significantly associated with amyloid load, both in preclinical [23] and prodromal populations [5, 6]. The current results are not in line with these previous findings, as we found neither a difference in awareness between amyloid-based groups nor a main effect of brain amyloidosis in our models. However, it is important to note that contrary to these previous studies, our models also accounted for tauopathy in addition to amyloidosis. Across the AD spectrum, tauopathy, more than amyloidosis, has been associated with cognitive and memory impairment, subsequent to lesion accumulation in medial temporal lobe (MTL) regions [51–54]. Over the course of AD, a current hypothesis suggests that tau spread is either induced or at least predisposed by amyloidosis [26, 55]. Recent results even demonstrated that tau lesions may mediate the cognitive relationship that can be observed with amyloid [56]. According to a cognitive hypothesis, unawareness is said to arise from a primary executive and/or memory deficit [57]. It could therefore be hypothesized that the presence of tauopathy in the transentorhinal cortices in early stages of AD could disturb episodic memory processes, leading to a progressive decline in the awareness of this growing deficit. In our data, this effect of tau lesions differed according to both the clinical stage and the amyloid load. As a matter of fact, in clinically normal (i.e., N) participants, we do not observe an effect of tau concentration in the EC at medium or high levels of amyloid, but a trending decline of awareness for high values of tau and low amyloidosis. On the other side, impaired participants with both high values of amyloid and tau demonstrated a lower awareness. These results may indicate two things. Firstly, the result that lower awareness was associated with tau burden without amyloid could support the hypothesis of a memory-related deficit in awareness due to tau, independently from amyloidosis. Secondly, the absence of effect in the control group for higher amyloid values and then the lower awareness in impaired participants with the presence of both biomarkers could indicate that the transition from SCD to anosognosia could occur later in the course of AD, when the cognitive impairment is sufficient to have a functional impact (i.e., when CDR ≥ 0.5). Considering previous research having demonstrated the presence of loss of awareness at early stages [21–23], one could suggest that this is the consequence of our grouping method, using the distinction between clinically normal (i.e., N) and impaired (i.e., I). Previous research demonstrated that tau accumulation starts in transentorhinal regions [55], the EC being among the first regions to be affected [55, 58–60], followed by a spread to the inferior temporal cortex (IT) [51, 58, 59] before progression to other regions in the brain through neuronal connections [55]. The significant three-way interaction of the clinical status and both biomarkers on the level of awareness in the EC but not in the IT model could advocate for an early impairment of awareness over the course of AD.

Moreover, in line with previous works [15, 61] and as mentioned above, our study used two types of awareness indexes, i.e., participant vs informant (participant-informant discrepancy index) and participant vs objective performance (participant-objective discrepancy index). To date, there is no consensus on how to optimally assess self-awareness. However, comparing different methods to measure awareness in the AD spectrum, Tondelli and colleagues [15] observed specific patterns of atrophy with some overlap, suggesting both specificity of methods but also common processes. More recently, Verfaillie and colleagues [61] demonstrated that increased memory concerns were related to increased amyloid burden. Importantly, this result was only observed when awareness was computed using a participant-objective discrepancy score, but not when using a participant-informant discrepancy score [61]. In contrast to Verfaillie, we observe the same pattern with both methods. That is, using both awareness indexes, we found a significant three-way interaction between status, amyloid, and tau burden in the entorhinal cortex but not in the inferior temporal cortex. As compared to the sample used in the study by Verfaillie et al., our participants were older and included a greater proportion of dementia stage, which could explain the different study outcomes. Consequently, the similar results observed using the two approaches in our study may be interpreted such that the informant’s complaints and participant’s memory deficits were converging, whereas the participant did not update his/her self-perception of cognitive difficulties, resulting in decreased self-awareness of memory.

In the study by Verfaillie and colleagues, they also observed an interaction between education and amyloid burden [61]. In contrast to these findings, we did not observe an interaction with education in the current sample (see Supplementary Table). This disparity could be explained either by a power issue (led by the increased number of terms in the model) or perhaps more likely, it might suggest that the addition of tau in our models, and the findings of a significant interaction effect between amyloid and tau, explained more of the variance. Similarly, the addition of tau in the models, as well as interactions with tau, may also explain the absence of a significant main effect of amyloid burden on the awareness that has been demonstrated in previous studies [5, 22, 23, 61]. However, this absence of effect can also be observed when simply comparing the groups (see Table 1), a result independent from the consideration of tau. These findings are in line with previous studies that have demonstrated a non-significant difference between awareness and amyloid in CN individuals [5, 6]. However, a recent study has observed that individuals with low awareness harbor increased levels of amyloid [23]. Several aspects could explain this variation across studies. For instance, the cross-sectional nature of the observations could obfuscate an essential dynamic factor by grouping together participants closer vs farther from putative progression. Indeed, if amyloid-positive CN could be considered as asymptomatic-at-risk individuals [29], we would have limited ability to predict when they would progress to clinical AD. When dichotomizing amyloid burden into positive and negative, an individual’s stage of progression is not taken into consideration, thus concealing the disparities and therefore obscuring any results. This limitation could be overcome not only by taking into account longitudinal change (e.g., in cognition, disease progression), but also by considering other factors that may be interwoven with the amyloid effect. For instance, a model controlling for certain covariates (e.g., age or the level of education, both important factors in individual risk for AD [29]) could be more comprehensive than a simple comparison. Therefore, the potential effect of amyloidosis on awareness could be overlooked if reducing the complexity of the amyloid measure (i.e., converting a continuous variable into binary), and then comparing groups.

Interestingly, in our data, we observed a significant difference between amyloid groups for the participant-objective score, but not for the participant-informant. This might indicate a higher sensitivity of the former, which could be explained by the fact that an informant’s score only reflects the partner’s subjective perception of the participant, and could thus be less accurate and precise as compared to objective cognitive measures. This distinction could be especially relevant at earlier stages, when subtle cognitive impairment does not necessarily interfere with activities of daily living.

### Limitations

This study has several limitations. Here, we explored awareness using both a participant-informant discrepancy based on daily functioning of memory and also a participant-objective discrepancy, which incorporated objective performance on an episodic memory task. We chose to focus on episodic memory in particular as this is one of the cognitive domains that is among the earlier to decline in AD [29, 62] and we also wanted to be consistent with previous research. Nonetheless, as loss of awareness may also arise from executive difficulties [57], a cognitive domain that is also declining early in AD [29, 63], it would be valuable to assess awareness via complaints related to this cognitive domain as well. In addition, there are several measures as well as approaches to compute awareness in literature, and standardization would be necessary to facilitate cross-study comparisons and generalization of results [4]. In this work, as in previous research [5], we choose to separate our sample based on functional abilities (i.e., using the CDR score). This could give us an over-schematic binary view of the awareness continuum masking the pattern of potential transition from heightened to declined awareness. Finally, we used a region of interest approach to assess the level of pathology (e.g., tau in the MTL [i.e., EC and IT] and amyloid in the whole brain). Future research should use an exploratory approach to see how awareness might be associated with increased pathology in other brain regions.

### Conclusion

In conclusion, using a cohort of both clinically normal and impaired individuals, lower awareness was only observed in clinically impaired individuals, and lower awareness was associated with higher measures of pathological burden. This provides further support that awareness measures may be important for clinical considerations. In order to extend these observations, longitudinal data will be crucial to assess the evolution of awareness across the disease trajectory.

## Supplementary Information

**Additional file 1: Figure S1.** Participant-Informant Awareness Index distributions across clinical groups. **Figure S2.** Objective-Subjective Awareness Index distributions across clinical groups. **Figure S3.** Self-Informant Awareness Index and AD Biomarkers (One- and Two-way effects). **Figure S4.** Awareness indexes and AD biomarkers distributions across clinical groups. **Table S1 Supplementary Models.** Relationship between Awareness, Education and AD Biomarkers. **Table S2 Supplementary Models.** Relationship between Awareness and AD Biomarkers for the whole sample.

**Additional file 2.**

## Data Availability

Requests for access to the original data should be addressed to the study sponsors. The ADNI data are available at http://adni.loni.usc.edu.

## Abbreviations

A+/A−: Amyloid positive/negative, i.e., AV45 PET above or below the 1.11 threshold
AD: Alzheimer’s disease
ADAS-COG: Alzheimer’s Disease Assessment Scale–Cognitive Subscale
ADNI: Alzheimer’s Disease Neuroimaging Initiative
APOE: Apolipoprotein
CDR: Clinical Dementia Rating
EC: Entorhinal cortex
ECog: Everyday Cognition
GDS: Geriatic Depression Scale
I: Impaired
IT: Inferior temporal cortex
MMSE: Mini-Mental State Examination
MSDR: Mean-to-standard deviation ratio
MTL: Medial temporal lobe
N: Normal
PET: Positron emission tomography
PVC: Partial-volume correction
RAVLT: Rey Auditory Verbal Learning Test
ROI: Region of interest
SCD: Subjective cognitive decline
SSA: Simple slope analyses
SUVr: Standard uptake volume ratio
