# Supplementary material for: Pathological correlates of impaired self-awareness of memory function in Alzheimer’s disease
Source: Alzheimers Res Ther. 2021 Jun 25;13:118. doi: 10.1186/s13195-021-00856-x (PMC8234669; doi:10.1186/s13195-021-00856-x)
Supplement: Supplementary file 1 — Additional file 1: Figure S1. Participant-Informant Awareness Index distributions across clinical groups. Figure S2. Objective-Subjective Awareness Index distributions across clinical groups. Figure S3. Self-Informant Awareness Index and AD Biomarkers (One- and Two-way effects). Figure S4. Awareness indexes and AD biomarkers distributions across clinical groups. Table S1 Supplementary Models. Relationship between Awareness, Education and AD Biomarkers. Table S2 Supplementary Models. Relationship between Awareness and AD Biomarkers for the whole sample. [file 13195_2021_856_MOESM1_ESM.pdf]

Pathological correlates of impaired self-awareness of memory function in Alzheimer's disease.

Supplementary Materials

Gagliardi, G., Kuppe, M., Lois, C., Hanseeuw, B., Vannini, P.,

for the Alzheimer's Disease Neuroimaging Initiative

Alzheimer's Research and Therapy

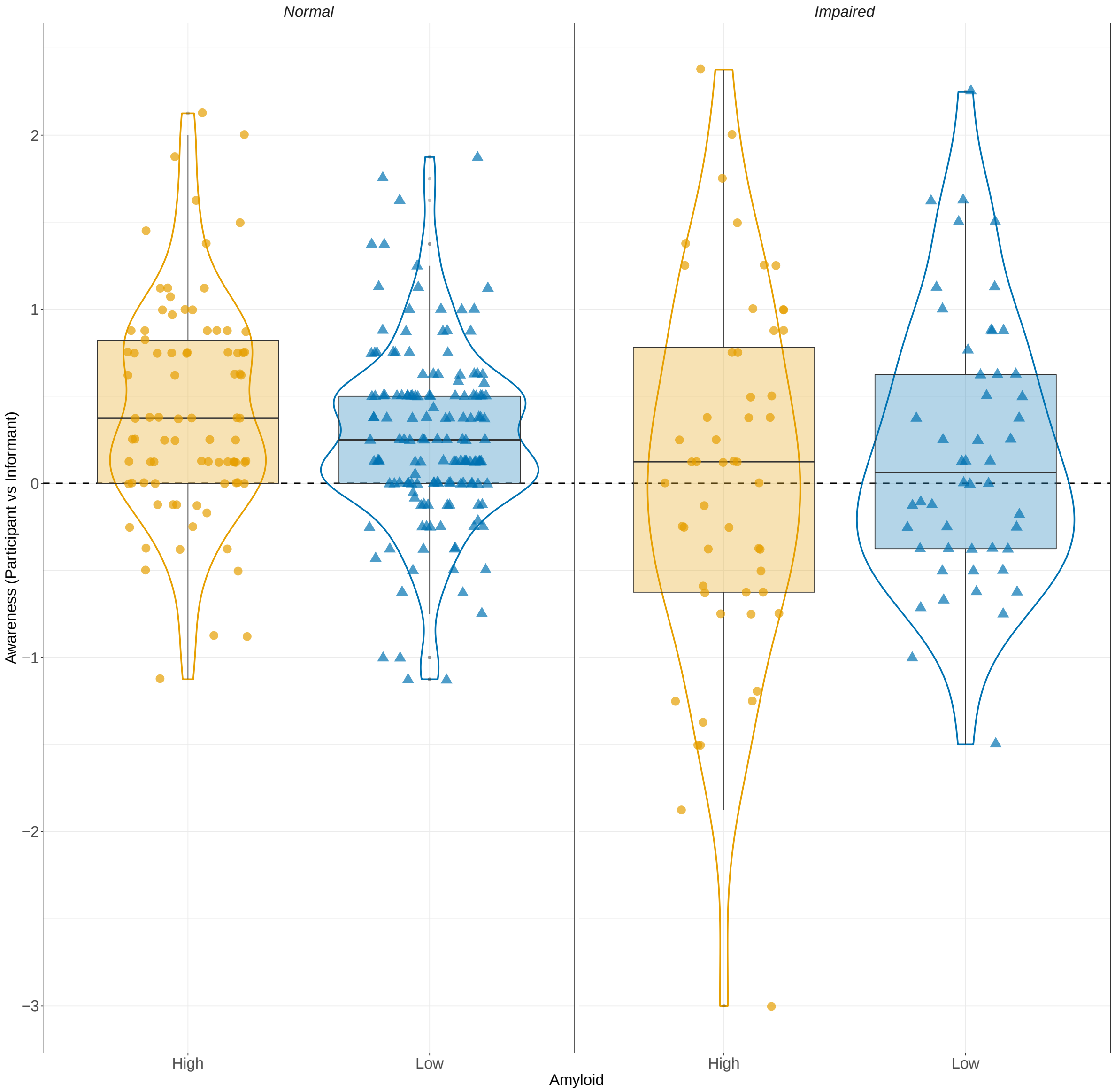

Boxplot of awareness (participant−informant index) in cognitively normal (left) and impaired (right) individuals.

Figure 1: Participant-Informant Awareness Index distributions across clinical groups

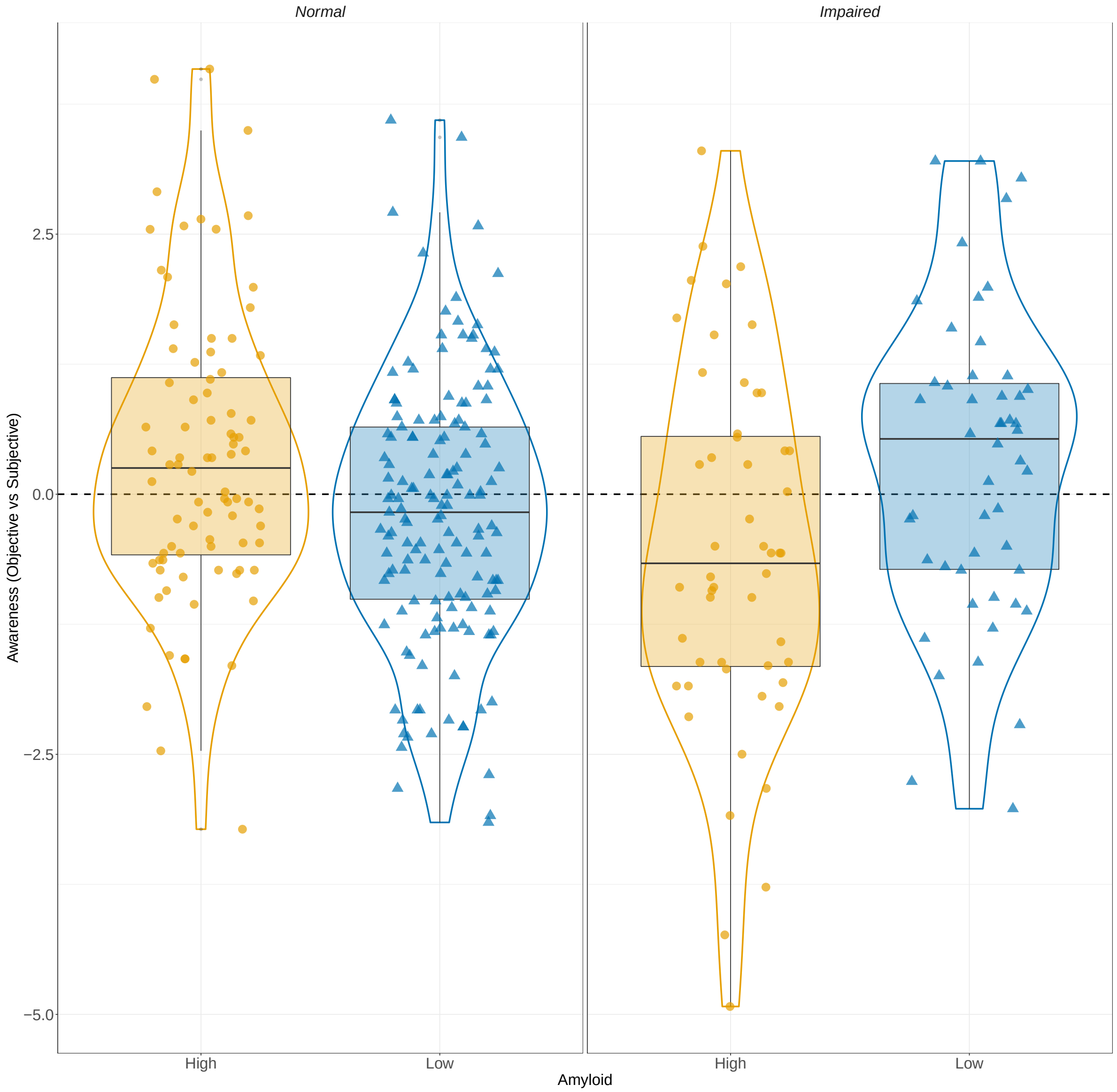

Boxplot of awareness (objective−subjective index) in cognitively normal (left) and impaired (right) individuals.

Figure 2: Objective-Subjective Awareness Index distributions across clinical groups

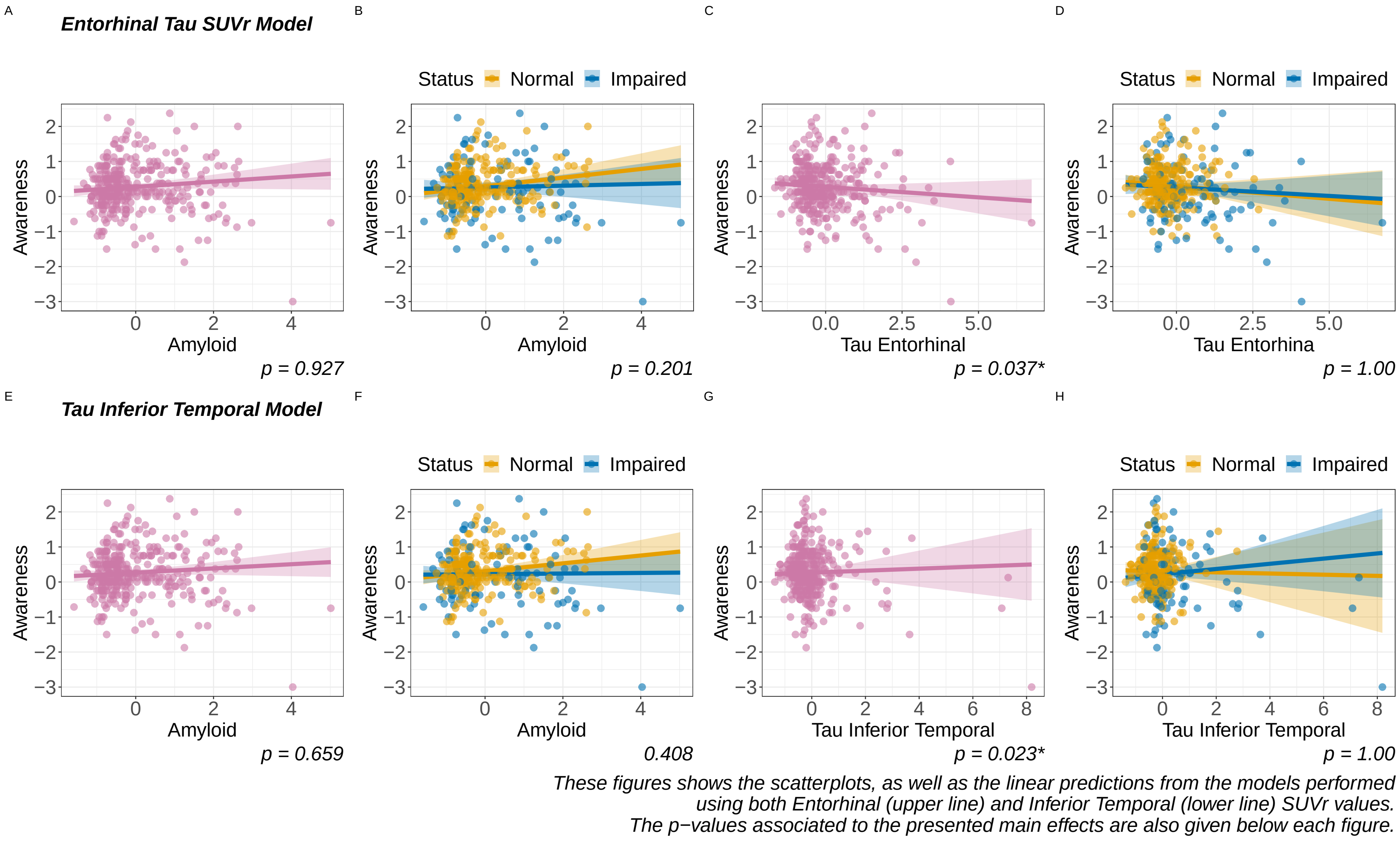

Figure 3: Self-Informant Awareness Index and AD Biomarkers (One- and Two-way effects)

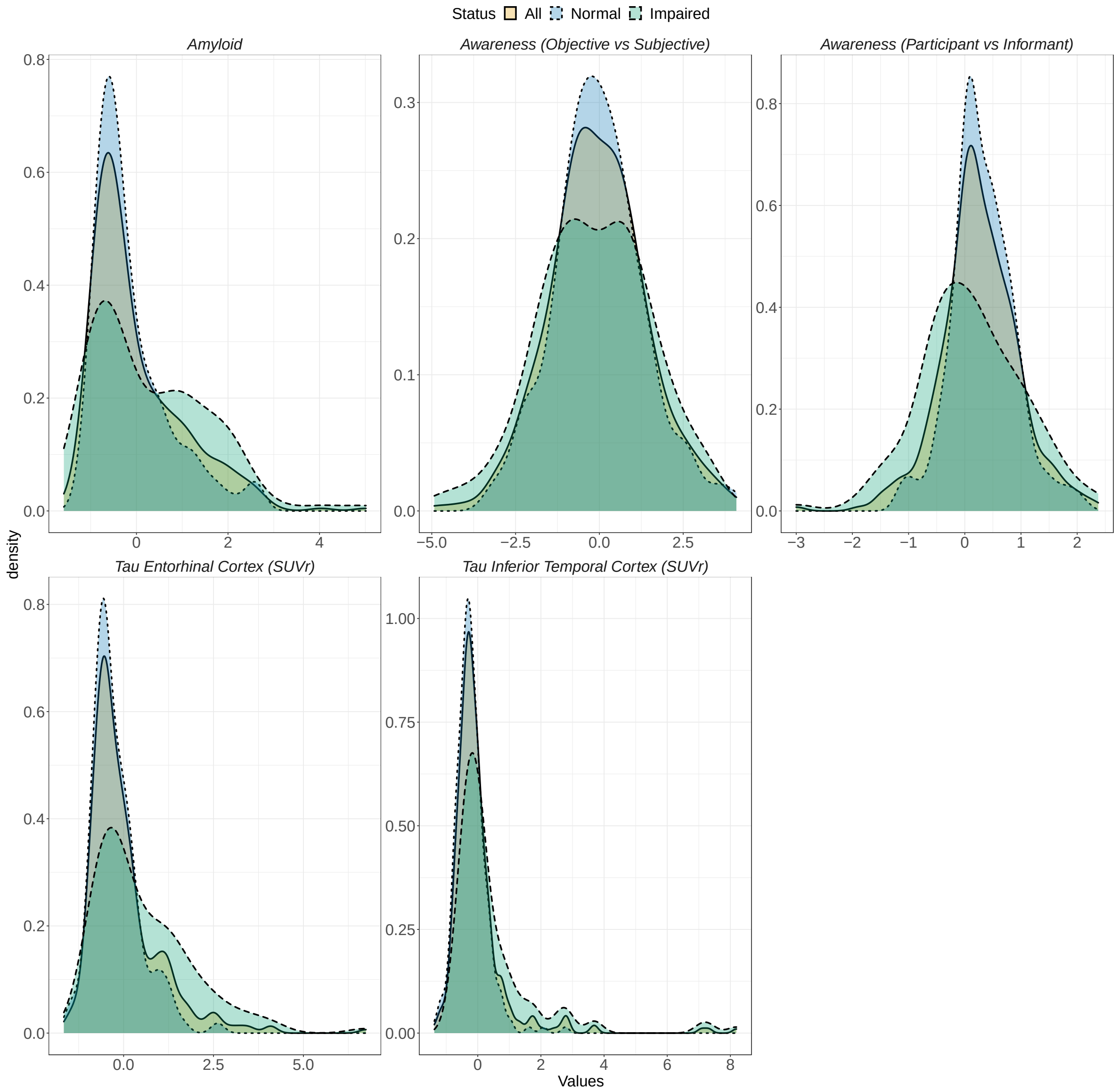

Density plots of awareness (objective−subjective and self−informantindexes) and AD biomarkers (amyloid and tau PET SUVr) in cognitively normal (blue) an

Figure 4: Awareness indexes and AD biomarkers distributions across clinical groups

Table 1: Supplementary Models: Relationship between Awareness, Education and AD Biomarkers

	Participants - Informants								Objective - Subjective							
	SUVr Tau Entorhinal				SUVr Tau Inferior Temporal				SUVr Tau Entorhinal				SUVr Tau Inferior Temporal			
Vars	Estimate	SD	ESs	P	Estimate	SD	ESs	P	Estimate	SD	ESs	P	Estimate	SD	ESs	P
(Intercept)	0.24	0.5			0.18	0.5			0.38	1.05			0.1	1.08		
Age	0	0.01	-0.001	1.000	0	0.01	0.001	1.000	-0.02	0.01	0.011	0.087	-0.02	0.01	0.009	0.111
Gender	-0.14	0.08	0.01	0.134	-0.14	0.08	0.01	0.144	-0.28	0.16	0.009	0.169	-0.29	0.17	0.01	0.153
Education	0.01	0.02	0	1.000	0.02	0.02	0	1.000	0.09	0.04	0	0.058	0.1	0.04	0	0.099
Clinical Status			0.001	0.779			0.004	0.264			0.002	0.677			0.005	1.000
Clinical StatusImpaired	0.29	0.51			0.58	0.51			0.95	1.08			1.41	1.09		
Amyloid	0.01	0.26	0	1.000	0.33	0.27	0.005	0.486	-0.47	0.54	0.002	0.559	-0.12	0.57	0	0.456
Tau	-0.61	0.27	0.016	0.030*	-1.17	0.4	0.026	0.037*	-0.29	0.57	0.001	0.057	-1.51	0.85	0.01	0.391
Education:Clinical Status			0.001	1.000			0.005	0.369			0.001	1.000			0.004	0.471
Education:Clinical StatusImpaired	-0.02	0.03			-0.04	0.03			-0.04	0.06			-0.08	0.06		
Education:Tau	0.03	0.02	0.013	0.083	0.07	0.02	0.027	0.006*	0.01	0.03	0	1.000	0.07	0.05	0.007	0.245
Education:Amyloid	0.01	0.02	0.001	1.000	-0.01	0.02	0.002	0.866	0.05	0.03	0.008	0.227	0.03	0.03	0.003	0.734
Clinical Status:Amyloid			0.005	0.173			0.008	0.166			0.012	0.034*			0.035	0.002*
Clinical StatusImpaired:Amyloid	-0.11	0.09			-0.14	0.08			-0.37	0.19			-0.61	0.18		
Clinical Status:Tau			0.001	1.000			0.004	1.000			0.001	1.000			0.005	0.531
Clinical StatusImpaired:Tau	0.06	0.09			0.14	0.13			0.1	0.19			0.34	0.27		
Amyloid:Tau	0.12	0.07	0	0.120	0.12	0.09	0	0.210	0.17	0.15	0	0.164	0.11	0.2	0	1.000
Clinical Status:Amyloid:Tau			0.024	0.012*			0.012	0.104			0.014	0.065			0.002	0.953
Clinical StatusImpaired:Amyloid:Tau	-0.22	0.08			-0.19	0.1			-0.36	0.17			-0.15	0.21		

Note:
SD = Standard Deviation, ESs = Effect Sizes (Cohen's f2), Amyloid = florbetapir PET SUVr, Tau = flortaucipir PET SUVr

Table 2: Supplementary Models: Relationship between Awareness and AD Biomarkers for the whole sample

	Participants - Informants								Objective - Subjective							
	SUVr Tau Entorhinal				SUVr Tau Inferior Temporal				SUVr Tau Entorhinal				SUVr Tau Inferior Temporal			
Vars	Estimate	SD	ESs	P	Estimate	SD	ESs	P	Estimate	SD	ESs	P	Estimate	SD	ESs	P
(Intercept)	0.3	0.46			0.55	0.46			0.78	0.96			0.98	0.98		
Age	0	0.01	0	1.000	0	0.01	0.001	1.000	-0.02	0.01	0.009	0.157	-0.02	0.01	0.01	0.128
Gender	-0.15	0.08	0.012	0.091	-0.14	0.08	0.011	0.113	-0.25	0.16	0.007	0.241	-0.24	0.16	0.007	0.288
Education	0	0.01	0	1.000	0	0.01	0	1.000	0.06	0.03	0.01	0.136	0.05	0.03	0.007	0.249
Amyloid	0.07	0.04	0.008	1.000	0.06	0.04	0.006	0.665	0.18	0.09	0.013	0.668	0.12	0.09	0.006	0.555
Tau	-0.09	0.04	0.013	0.001*	0.01	0.06	0	<0.001*	-0.12	0.09	0.005	0.013*	-0.02	0.13	0	0.019*
Amyloid:Tau	-0.09	0.03	0.032	0.003*	-0.1	0.02	0.048	<0.001*	-0.2	0.06	0.035	0.002*	-0.12	0.05	0.016	0.046*

Note:
SD = Standard Deviation, ESs = Effect Sizes (Cohen's f2), Amyloid = florbetapir PET SUVr, Tau = flortaucipir PET SUVr
